# Maternal exercise conveys protection against NAFLD in the offspring via hepatic metabolic programming

**DOI:** 10.1038/s41598-020-72022-6

**Published:** 2020-09-22

**Authors:** Inga Bae-Gartz, Philipp Kasper, Nora Großmann, Saida Breuer, Ruth Janoschek, Tobias Kretschmer, Sarah Appel, Lisa Schmitz, Christina Vohlen, Alexander Quaas, Michal R. Schweiger, Christina Grimm, Axel Fischer, Nina Ferrari, Christine Graf, Christian K. Frese, Sonja Lang, Münevver Demir, Christoph Schramm, Gregor Fink, Tobias Goeser, Jörg Dötsch, Eva Hucklenbruch-Rother

**Affiliations:** 1 Department of Pediatrics and Adolescent Medicine, University of Cologne, Faculty of Medicine and University Hospital Cologne, Robert-Koch Str. 16, Building 44a, 50931 Cologne, Germany; 2 Department of Gastroenterology and Hepatology, University of Cologne, Faculty of Medicine and University Hospital Cologne, Cologne, Germany; 3 Department of Pathology, University Hospital of Cologne, Cologne, Germany; 4 Translational Epigenetics and Tumor Genetic, University Hospital of Cologne, Cologne, Germany; 5 iCoder, Potsdam, Germany; 6 Cologne Center for Prevention in Childhood and Youth / Heart Center Cologne, University Hospital of Cologne, Cologne, Germany; 7 Institute of Movement and Neuroscience, Department of Movement and Health Promotion, German Sport University, Cologne, Germany; 8 Proteomics Core Facility, CECAD Research Center, University Hospital of Cologne, Cologne, Germany; 9 Max-Planck-Unit for the Science of Pathogens, Charité University Medicine Berlin, Berlin, Germany; 10 Department of Medicine, University of California San Diego, La Jolla, CA USA; 11 Department of Hepatology and Gastroenterology, Charité Campus Mitte and Campus Virchow Clinic, Charité University Medicine Berlin, Berlin, Germany

**Keywords:** Biomarkers, Gastroenterology, Medical research, Molecular medicine, Pathogenesis

## Abstract

Maternal exercise (ME) during pregnancy has been shown to improve metabolic health in offspring and confers protection against the development of non-alcoholic fatty liver disease (NAFLD). However, its underlying mechanism are still poorly understood, and it remains unclear whether protective effects on hepatic metabolism are already seen in the offspring early life. This study aimed at determining the effects of ME during pregnancy on offspring body composition and development of NAFLD while focusing on proteomic-based analysis of the hepatic energy metabolism during developmental organ programming in early life. Under an obesogenic high-fat diet (HFD), male offspring of exercised C57BL/6J-mouse dams were protected from body weight gain and NAFLD in adulthood (postnatal day (P) 112). This was associated with a significant activation of hepatic AMP-activated protein kinase (AMPK), peroxisome proliferator-activated receptor alpha (PPARα) and PPAR coactivator-1 alpha (PGC1α) signaling with reduced hepatic lipogenesis and increased hepatic β-oxidation at organ programming peak in early life (P21). Concomitant proteomic analysis revealed a characteristic hepatic expression pattern in offspring as a result of ME with the most prominent impact on Cholesterol 7 alpha-hydroxylase (CYP7A1). Thus, ME may offer protection against offspring HFD-induced NAFLD by shaping hepatic proteomics signature and metabolism in early life. The results highlight the potential of exercise during pregnancy for preventing the early origins of NAFLD.

## Introduction

Non-alcoholic fatty liver disease (NAFLD) is becoming a major health problem and has emerged as the leading cause of chronic liver disease, even in overweight children, adolescents and young adults^1,2^. Since exercise and weight loss currently represent the only approved treatment options for NAFLD, identification of new therapeutic targets for the treatment and prevention of NAFLD are subject of intensive research.

The perinatal environment is a critical window for offspring health during later life. The Barker theory proposes that alterations in early life environment shape the metabolic phenotype and individuals’ risk of developing metabolic disorders in later life^3,4^. Beneficial health behavior of the mother, such as maternal exercise (ME), during this period has been shown to improve metabolic health of offspring^5–7^. However, only 9–15% of pregnant women meet the current physical activity recommendations of moderate-intensity activity of 150 min per week^5^. Focusing on the underlying mechanisms, the beneficial effects of ME on offspring have previously been attributed to e.g. altered hormone signaling, epigenetic effects or modifications in organ specific gene expression during the prenatal and/or early postnatal period^7–11^.

Regarding the pathogenesis of NAFLD, there is growing evidence that different environmental exposures in utero and during early life lead to persistent changes in the hepatic metabolism^6,12,16^. While an exposure to an unfavorable perinatal environment, mediated by maternal obesity or gestational diabetes, may contribute to an increased individual’s susceptibility to NAFLD, preventive interventions such as ME during critical periods of developmental programming seems to be able to reduce the susceptibility to and severity of NAFLD through direct effects on the liver and indirect effects via metabolic programming^13^. However, the underlying molecular mechanism remains poorly understood.

In order to gain deeper insights into the potential protective effects of ME on the development of hepatic disorders such as high-fat diet induced NAFLD, we investigated how ME during pregnancy affects offspring hepatic proteome and molecular target factors of hepatic lipid and glucose homeostasis at organ *programming peak* in early life (postnatal day (P) 21).

## Results

### Maternal exercise has no effect on gestational weight gain or serum leptin and insulin levels

We first determined maternal body weight before and during pregnancy and quantified the amount of exercise. There was no significant difference in maternal weight gain during pregnancy (Supplementary Fig. 1B). The average distance of voluntary wheel running was around 10 km/day for the first days of gestation (Supplementary Fig. 1A). When the pregnant mice approached delivery, the average distance of voluntary wheel running was around 400 m/day. There were no significant effects on maternal non fasted serum leptin or insulin levels at gestational day (G)16 (Supplementary Fig. 1C,D). Portions of dams and offspring characteristics of this cohort of mice have been reported previously^14^. For convenience, pertinent data are summarized in Supplementary Fig. 1.

**Figure 1:**
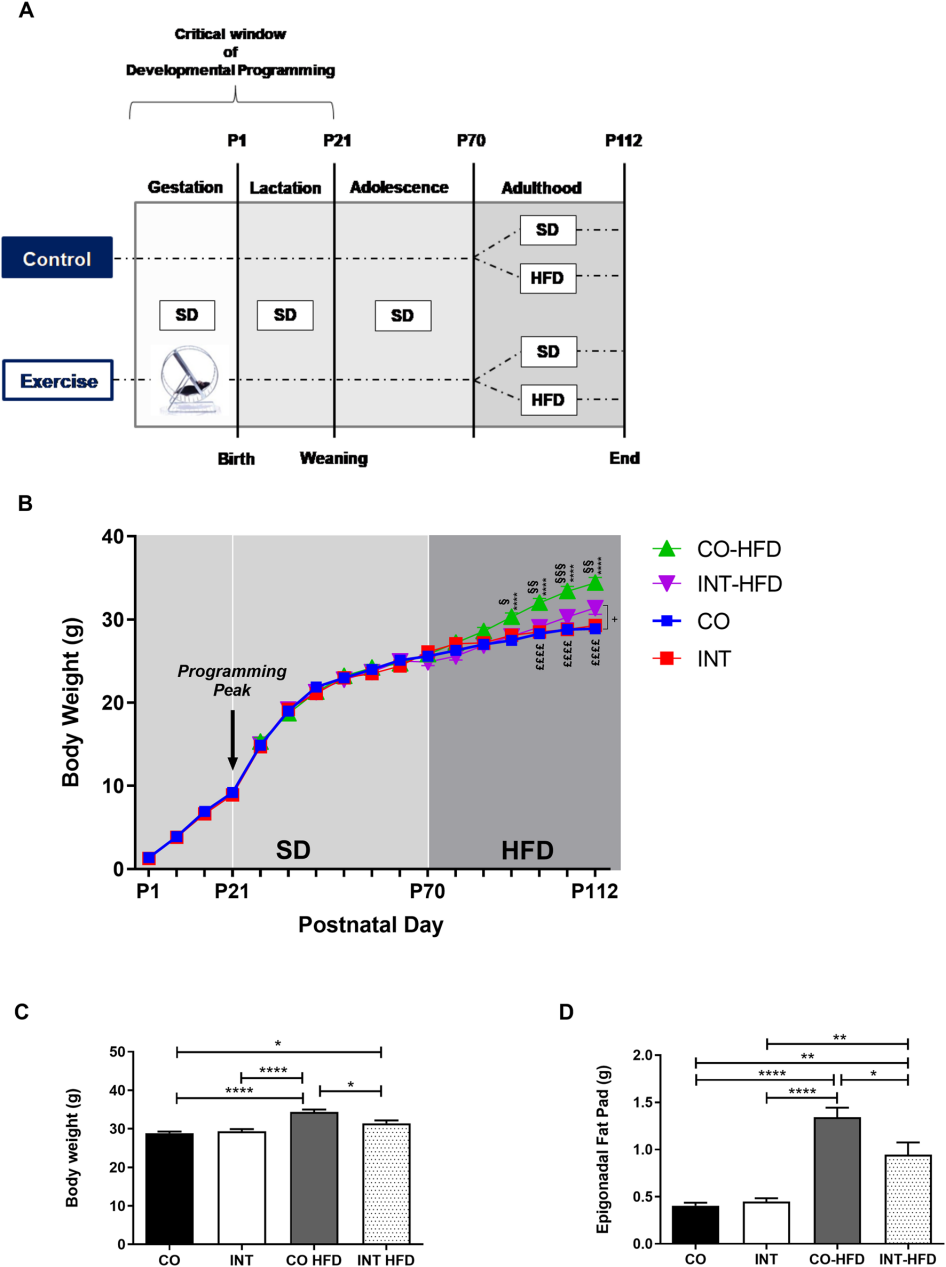
Maternal gestational exercise protects offspring from HFD-induced body weight gain (**A**) Experimental design. (**B**) Offspring body weight gain (§ Differences between CO-HFD and INT-HFD, * Differences between CO-HFD and CO, £ Differences between CO-HFD and INT, + Difference between CO and INT-HFD). (**C**) Total body weight at P112. (**D**) Epigonadal fat pad weight at P112. [CO (n = 28), INT (n = 14), CO-HFD (n = 33), INT-HFD (n = 9)]. Mean ± SEM;*^/+/§/£^*p* < 0.05, **^/++/§§/££^*p* < 0.01, ***^/+++/§§§/£££^*p* < 0.001, ****^/++++/§§§§/££££^*p* < 0.0001. *CO* control, *INT* intervention, *HFD* high fat diet, *SD* standard diet, *g* gram, *P* postnatal day.

### Gestational exercise protects offspring against HFD-induced weight gain

To determine the effects of ME on offspring body weight gain, body weight was measured daily during the lactation period (P1-P21) and once weekly from P21 to P112. There was no significant difference in offspring body weight during the lactation period, at P21 and up to P70. Epigonadal-fat-pad weight and serum leptin and insulin levels revealed no significant differences at P21 (Supplementary Fig. 2). Upon the start of HFD-feeding at P70, body weight increased rapidly in the sedentary control (CO-HFD) group, while offspring of exercised dams (INT-HFD) remained significantly lighter at all times (Fig. 1B,C, *p* < 0.01). At P112 CO and INT-offspring on standard chow diet did not differ with regard to epigonadal adipose tissue mass, whereas INT-HFD offspring was protected against the significant HFD-induced increase in adipose tissue mass as seen in CO-HFD offspring (Fig. 1D, *p* < 0.05).

**Figure 2:**
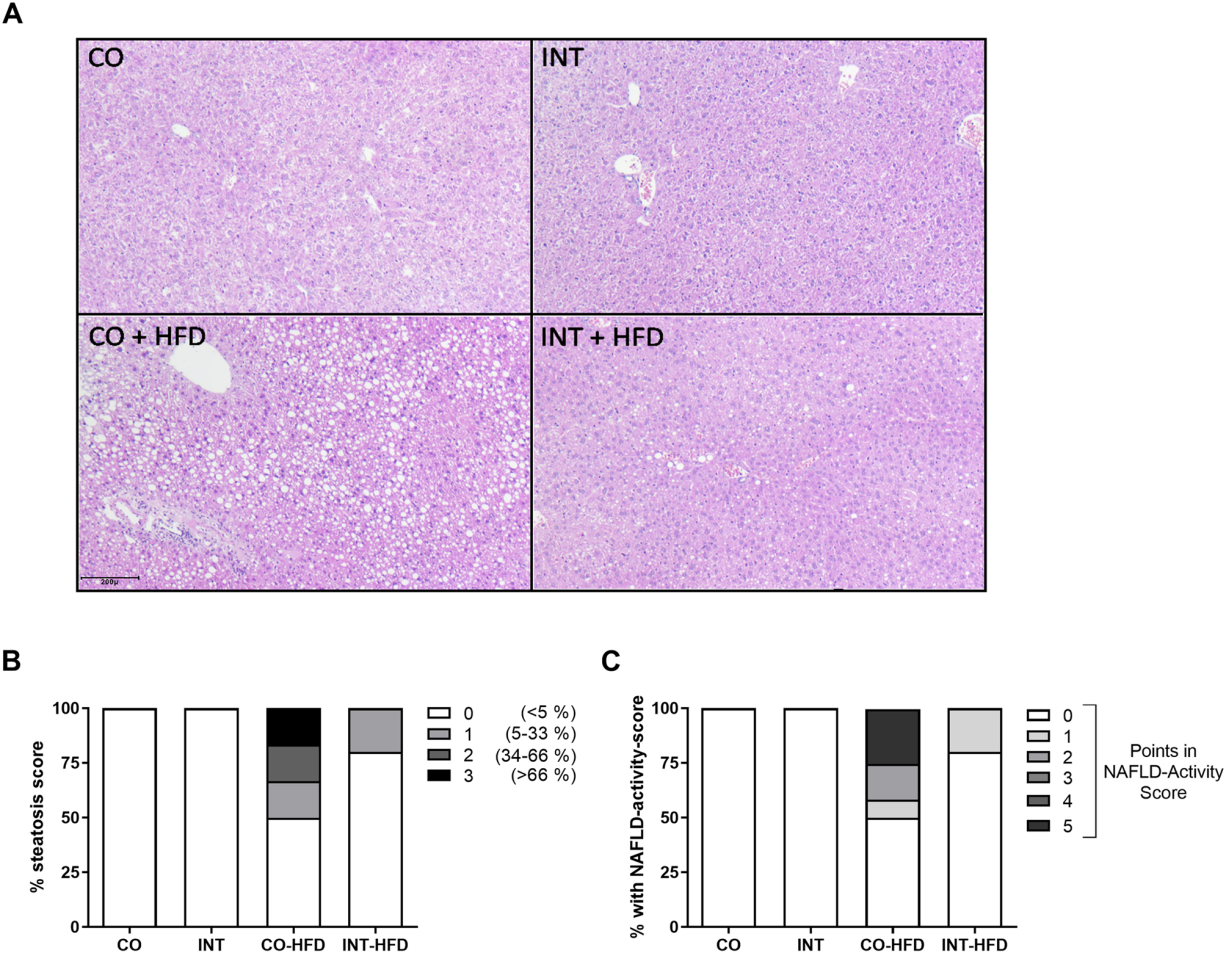
Maternal exercise during pregnancy protects offspring from NAFLD in later life. (**A**) Histological hepatic steatosis in 4-month old male offspring (P112) by H&E staining. Magnification: 20×. (**B**) Percentage of steatosis, scored as 0 (< 5%); 1 (5–33%); 2 (34%-66%) and 3 (> 66%) percentage of hepatocytes containing lipid droplets. (**C**) Liver-biopsy assessment of NAFLD-activity score with average score. CO (n = 6), INT (n = 5), CO-HFD (n = 12), INT-HFD (n = 6). *CO* control, *INT* intervention, *NAFLD* non -alcoholic fatty liver disease, *HFD* high fat diet, *H&E* hematoxylin and eosin staining.

### Maternal exercise during pregnancy prevents offspring from HFD-induced hepatic steatosis

To evaluate the effects of ME during pregnancy on hepatic lipid content, liver histomorphology was analyzed at P112. CO-HFD offspring showed the strongest fat deposition (grade 1–3), whereas the offspring of exercised mothers were mainly protected against hepatic steatosis (Fig. 2). In 3 of 12 CO-HFD subjects, HFD-feeding induced histological features of non-alcoholic steatohepatitis (NASH), including steatosis, inflammation and hepatocellular ballooning after only six weeks of HFD-challenge (Fig. 2B,C). Thus, six weeks of HFD are sufficient to provoke NAFLD/NASH in offspring.

### Gestational exercise induces a characteristic hepatic proteome signature in offspring

**Figure 3:**
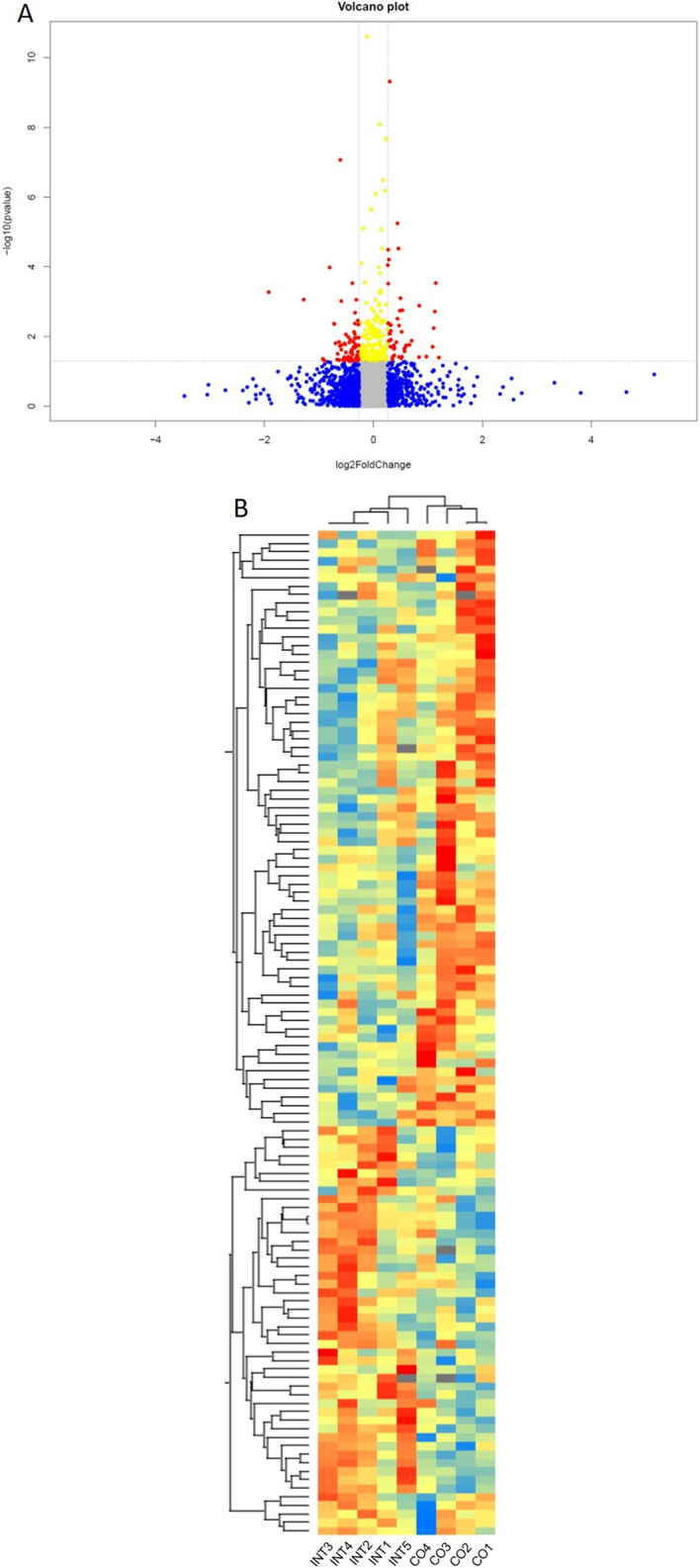
Maternal gestational exercise induces a distinct metabolic signature in the liver proteome of INT offspring at P21. (**A**) Volcano plot of univariate statistical analysis results from liver tissue of CO- and INT-offspring. A volcano plot based on fold change (Log_2_) and *P* value (− Log_10_) of all proteins identified in both groups. Red dots indicate proteins that showed statistically significant changes (N = 118). (**B**) Heatmap of unsupervised 2-dimensional hierarchical clustering of the proteome profile of significantly altered proteins. The normalized Z-Score of protein abundance is depicted by a pseudocolor scale with blue showing positive expression, yellow showing equal expression and red negative expression compared with the values of each protein. Visual inspection of the heatmap demonstrates the ability of these proteins to distinguish between offspring of exercised or sedentary dams. CO (n = 4), INT (n = 5). *CO* control, *INT* intervention, *log* logarithm.

To get comprehensive insights into offspring liver protein signatures and possible origins of NAFLD, P21-liver samples were subjected to proteomic analysis. A total of 3,876 proteins were identified and quantified with a FDR of 0.01 (Supplementary Table 3). Among these, the abundance of 118 proteins, which are listed in the Supplementary Table 4, was significantly altered based on the criteria that both the fold change was ≥ 1.2 and t-test showed *p* ≤ 0.05 in two compared groups (Fig. 3A). Hierarchical clustering of the 118 proteins revealed a heat map of the proteomic differences from four CO-offspring and five INT-offspring (Fig. 3B). The heatmap indicates that the two different maternal conditions during pregnancy (exercise vs. no-exercise) were able to induce a distinct hepatic protein expression pattern in the offspring (Fig. 3B).

**Figure 4:**
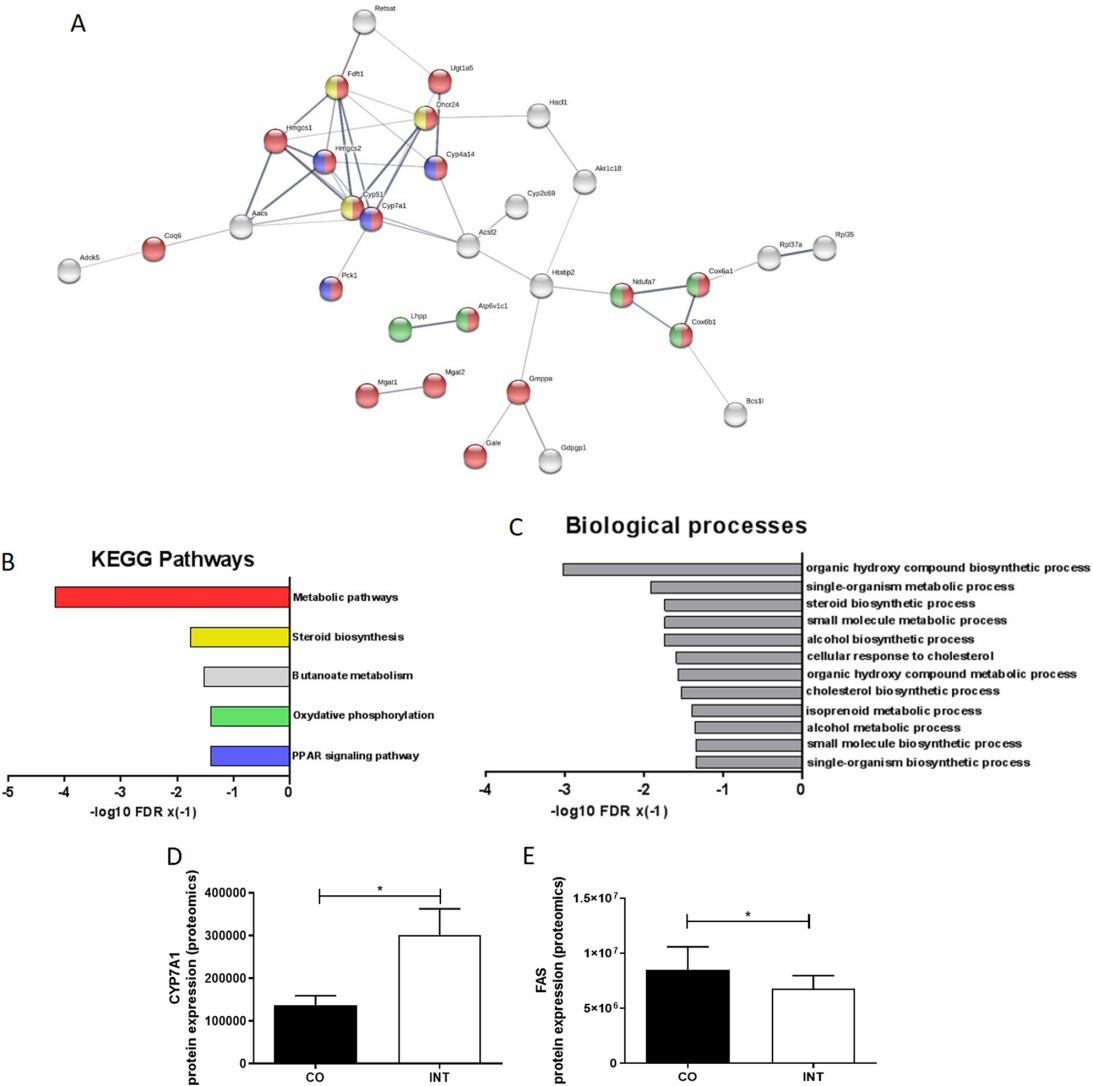
Influences of maternal exercise on hepatic proteome analysis of CO and INT offspring in early life. (**A**) Analysis of the biological functions of differential expressed proteins via STRING database (https://string-db.org) to yield gene ontology annotation terms for KEGG pathways and biological processes; network nodes represent proteins. (**B**) KEGG Pathways and (**C**) biological process-based categories of liver proteins that displayed significantly changed levels among CO-and INT-offspring at P21. (**D**) CYP7A1 protein expression. (**E**) FAS protein expression at P21. CO (n = 4), INT (n = 5). *CO* control, *INT* intervention, *KEGG* Kyoto Encyclopedia of Genes and Genomes, *P* postnatal day.

To analyze the biological functions or involved pathways of differential expressed proteins, we performed analysis via STRING-database (https://string-db.org) to yield gene ontology annotation terms for Kyoto Encyclopedia of Genes and Genomes (KEGG)—pathways and biological processes (GO-Terms). (Fig. 4A–C). KEGG—pathway analysis showed significant influences of ME on offspring hepatic “metabolic pathways”, “steroid biosynthesis”, “oxidative phosphorylation” and “PPAR signaling” (Fig. 4B). GO-Terms confirmed the influence of ME on hepatic metabolic processes in the offspring (Fig. 4C). Of note, the most regulated protein among the differentially expressed proteins was CYP7A1 (Cholesterol 7 alpha-hydroxylase), a key regulator of hepatic cholesterol and bile acid metabolism^15,16^. CYP7A1 protein expression levels were significantly increased in INT-offspring (Fig. 4D) while FAS (Fatty acid synthase), a protein involved in catalyzing fatty acid synthesis, revealed decreased protein expression levels in INT-offspring (Fig. 4E).

### Maternal exercise activates offspring AMPK-ACC-CPT1a axis

**Figure 5:**
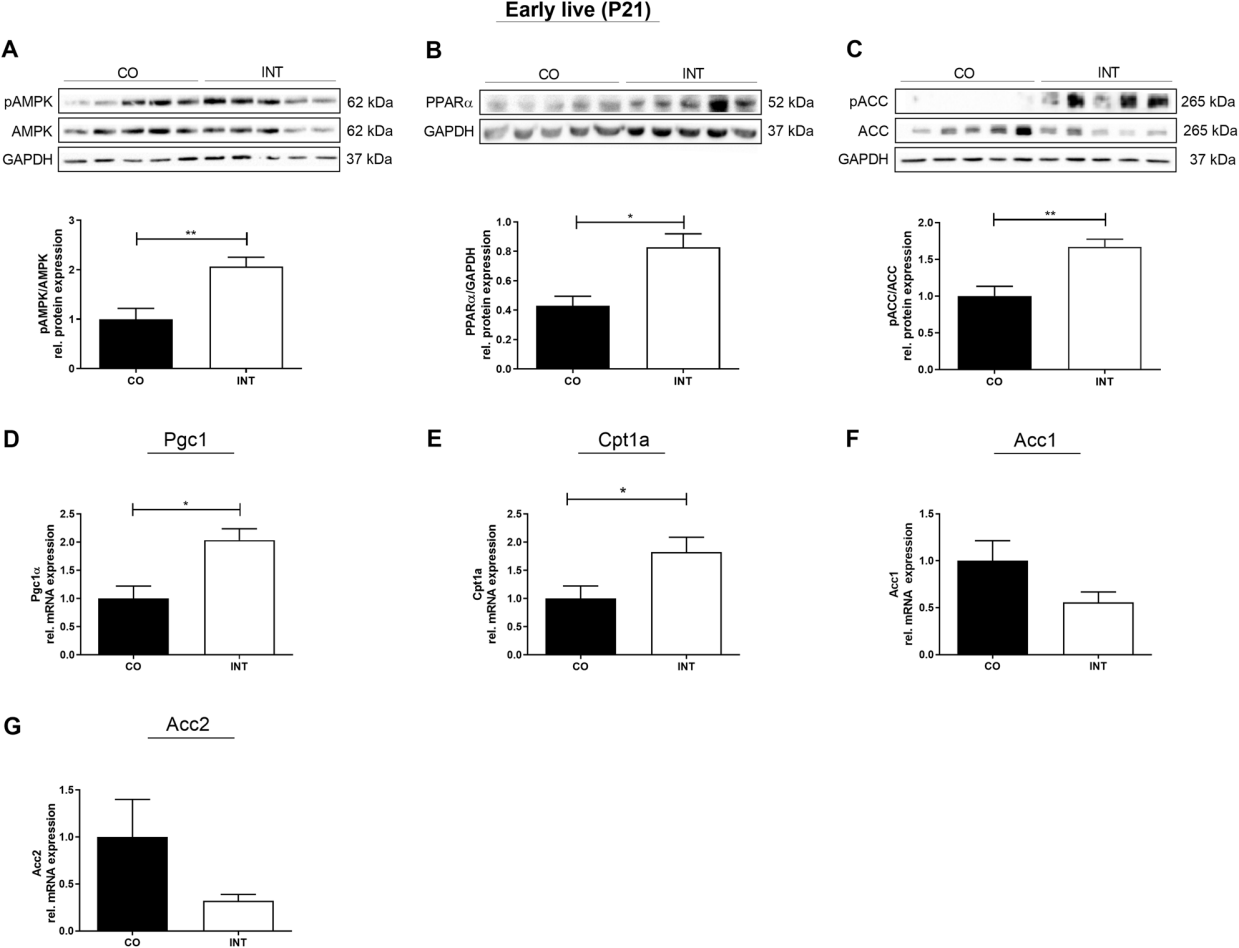
Effects of maternal exercise during pregnancy on offspring liver energy sensing and on AMPK signaling in early life. (**A**) pAMPK/AMPK protein expression. (**B**) PPARα protein expression. (**C**) pACC/ACC protein expression. (**D**) Pgc1α mRNA expression. (**E**) Cpt1a mRNA expression. (**F**) Acc1 mRNA expression. (**G**) Acc2 mRNA expression. Representative immunoblots are presented above the respective graph. Immunoblots: CO (n = 5), INT (n = 5). Uncropped images of original blots are shown in Supplementary Fig. 6. mRNA Expression: CO (n = 5), INT (n = 7). Mean ± SEM; *p < 0.05, **p < 0.01. *CO* control, *INT* intervention, *P* postnatal day, *AMPK* adenosine monophosphate-activated protein kinase, *PPARα* peroxisome proliferator-activated receptor alpha, *ACC* acetyl-CoA carboxylase, *GAPDH* Glycerinaldehyd-3-phosphate-dehydrogenase, *Pgc1α* peroxisome proliferator-activated receptor gamma coactivator 1-alpha, *Cpt1a* carnitine palmitoyltransferase 1A.

To analyze specific effects of ME on hepatic metabolism we then focused on key regulators of hepatic energy metabolism, such as AMP-activated protein kinase (AMPK), peroxisome proliferator-activated receptor alpha (PPARα) and PPAR coactivator-1 alpha (PGC1α), which are known to mediate exercise effects^17–20^. We initially determined protein levels of phosphorylated (p)AMPK and its downstream targets ACC (acetyl-CoA carboxylase) and PPARα by western blot. AMPK regulates hepatic lipid metabolism through the phosphorylation of ACC and activation of PPARα that leads to an inhibition of hepatic de novo lipogenesis and stimulation of fatty acid oxidation, representing key aspects in NAFLD pathogenesis^18–24^. Here we found a significant increase of pAMPK (*p* < 0.01), pACC (*p* < 0.01) and PPARα (*p* < 0.05) protein levels in INT-offspring compared to CO-offspring at P21 (Fig. 5A–C). Accordingly, mRNA-expression levels of ACC 1 and 2 tended to be downregulated (Fig. 5F,G). Since AMPK is also known to increase fatty acid oxidation by activation of *Cpt1a* (Carnitine palmitoyltransferase-1), we next focused on this marker and found a strong increase in *Cpt1a* mRNA-expression level in INT-offspring at P21 (Fig. 5E, *p* < 0.05)^17^. Thus, our findings suggest a favorable programming of hepatic lipid metabolism in INT-offspring in early life marked by an activation of the AMPK- ACC–CPT1a axis. Intriguingly, in later life (P112) after offspring were exposed to HFD, an ongoing significant upregulation of pAMPK in the NAFLD-protected INT-HFD offspring was still observed, compared to CO-HFD offspring (Supplementary Fig. 3A, *p* < 0.01).

To further elucidate the effects of ME on offspring metabolism, we next investigated Pgc1α, also known as PPARGC1alpha, which represents another important sensor of energy metabolism, key factor in mediating exercise training effects, and an interaction partner of AMPK^18–22,25,26^. Here, we also found significantly increased mRNA-expression levels of Pgc1α in INT-offspring at P21 compared to CO (Fig. 5D, *p* < 0.05).

To analyze if the upregulation was due to epigenetic modifications induced by ME, we assessed the DNA-methylation of the Pgc1a promoter by bisulfite conversion, which converts unmethylated cytosines to uracil, followed by PCR-amplification and massive parallel sequencing at a coverage of at least 450 × per CpG (5′-Cytosine-phosphate-Guanin-3′ nucleotide sequence). To this end, we analyzed the methylation levels of all seven CpGs residing in a 1 kb region upstream of the transcription start site (Supplementary Fig. 4A). However, there were no significant differences in methylation levels between CO- and INT-group at P21 (Supplementary Fig. 4B). As PGC1α also mediates mitochondrial biogenesis and ‘oxidative phosphorylation’ was significantly influenced in the proteomic screen, we next analyzed Tfam (Mitochondrial transcription factor A) mRNA-expression. Tfam mRNA-levels did not differ between CO- and INT-offspring at P21 (Supplementary Fig. 2E).

### Effects of maternal exercise during pregnancy on offspring glucose and insulin metabolism

**Figure 6:**
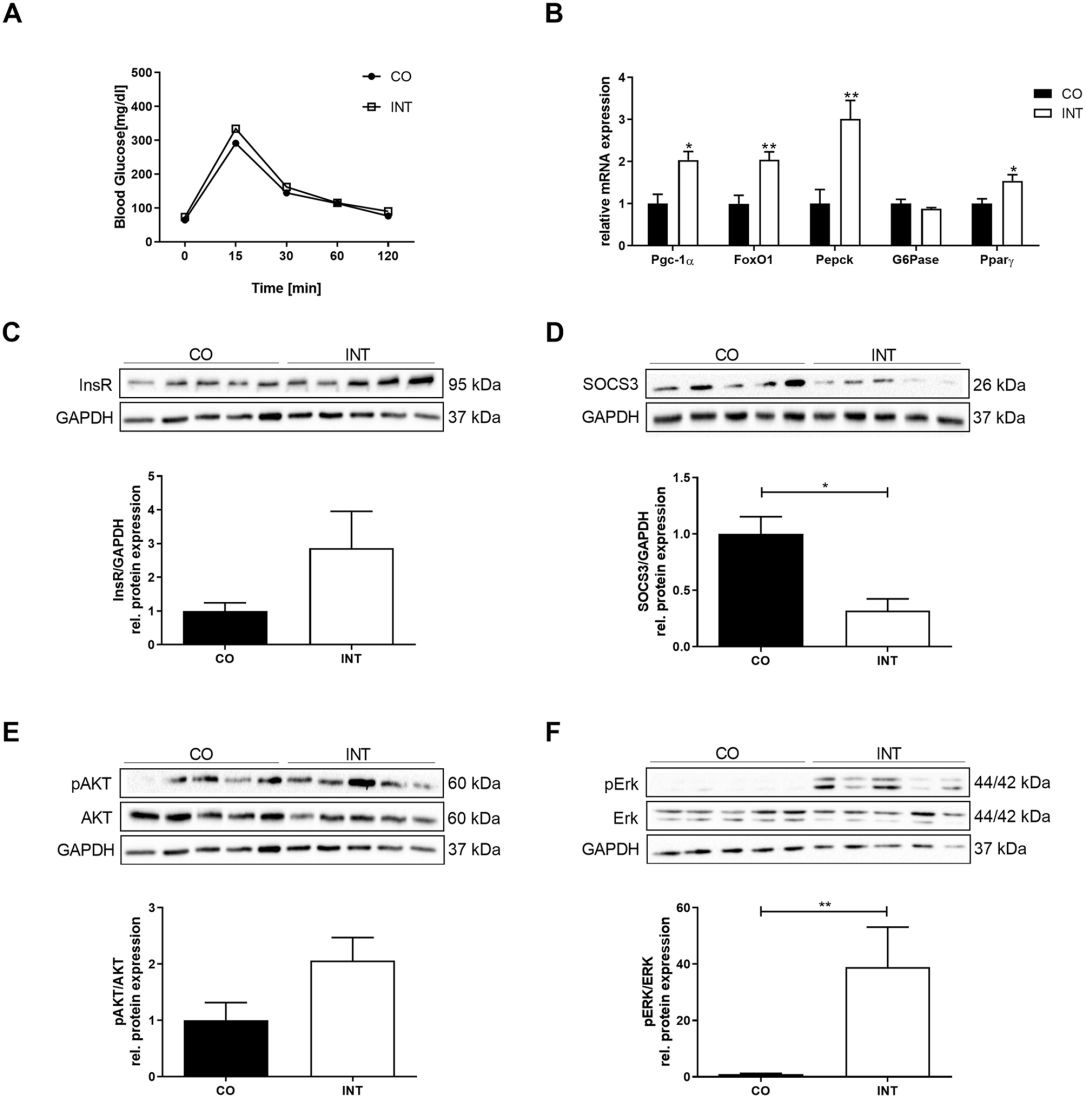
Impact of maternal gestational exercise on hepatic glucose metabolism in offspring at P21. (**A**) Intraperitoneal glucose tolerance test (i.p. GTT) at P21 [CO (n = 12), INT (n = 8)]. (**B**) Assessment of regulators of hepatic glucose metabolism (Pgc1α, FoxO1, Pepck, G6Pase, Pparƴ) by qPCR [CO (n = 5), INT (n = 5–7)]. (**C**–**F**) Assessment of indicators of insulin signaling at P21 using immunoblots: (**C**) INS-R, (**D**) SOCS-3, (**E**) pAKT/AKT, (**F**) pERK/ERK [CO (n = 5), INT (n = 5)]. Representative immunoblots are presented above the respective graphs. Uncropped images of original blots are shown in Supplementary Fig. 6. Mean ± SEM; *p < 0.05, **p < 0.01. *CO* control, *INT* intervention, *Ins-R* Insulin receptor, *SOCS-3* suppressor of cytokine signaling 3, *erk* extracellular signal-regulated kinases, *GAPDH* Glycerinaldehyd-3-phosphate-dehydrogenase.

To investigate whether the upregulation of different energy regulators was also associated with changes in offspring glucose metabolism at P21, we determined serum insulin levels and performed glucose tolerance testing, which both revealed no significant differences between CO- and INT-offspring even though insulin levels tended to be lower in INT-offspring (Fig. 6A, Supplementary Fig. 2C). Next, we determined hepatic protein expression of the INS-R (Insulin receptor) and activation of its downstream target AKT (Fig. 6C,E) to get insights into hepatic insulin signaling. Both showed a tendency towards an increase in INT-offspring paralleled by significantly increased mRNA-levels of insulin target genes *Foxo1* (Forkhead box O1) and *Pparγ* (Fig. 6B,C,E, *p* < 0.5 and *p* < 0.01). Furthermore, we detected a strong activation of hepatic ERK1/2 (Extracellular-signal-regulated kinase) in INT-offspring compared to controls (Fig. 6F, *p* < 0.01). To assess markers of hepatic insulin resistance, SOCS3 (suppressor of cytokine signaling 3) protein expression was determined and showed a clear reduction in INT-offspring at P21 (Fig. 6D, *p* < 0.05)^27^. Additionally, we determined *Pepck* (Phosphoenolpyruvat-Carboxylase) mRNA-levels which revealed a significant increase in INT-offspring, while *G6pase* (Glucose-6-Phosphatase) did not differ between the groups (Fig. 6B).

### Effects of maternal gestational exercise on offspring cholesterol metabolism

**Figure 7:**
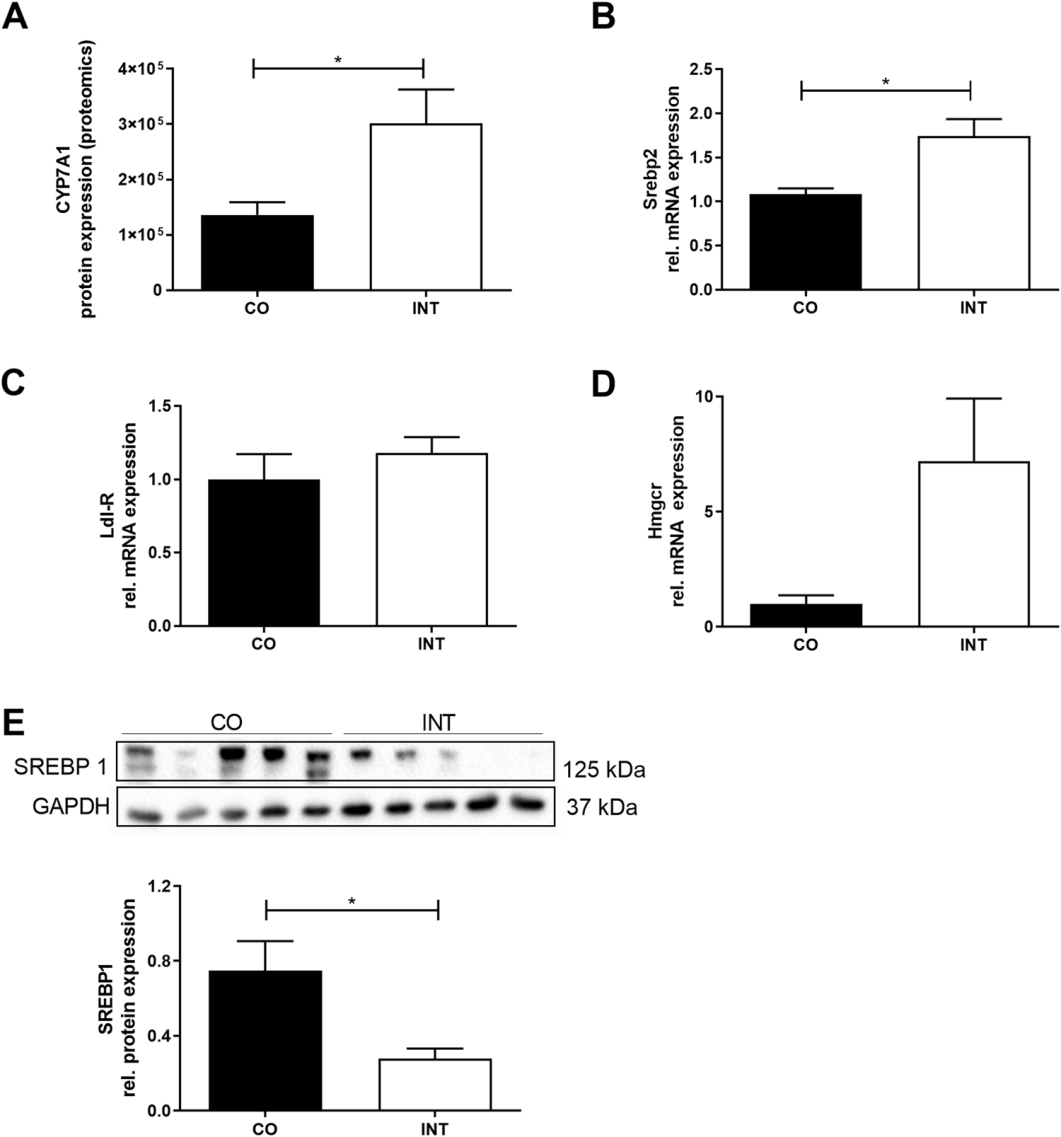
Influences of maternal exercise on offspring cholesterol at P21. (**A**) CYP7A1 protein expression [CO (n = 4), INT (n = 5)]. (**B**) Srebp2 mRNA expression [CO (n = 11), INT (n = 7)]. (**C**) Ldl-R mRNA expression [CO (n = 11), INT (n = 8)]. (**D**) Hmgcr mRNA expression [CO (n = 5), INT (n = 7)]. (**E**) SREBP1 protein expression [CO (n = 5), INT (n = 5)]. Representative immunoblots are presented above the respective graph. Uncropped images of original blots are shown in Supplementary Fig. 6. Mean ± SEM; *p < 0.05. *CO* control, *INT* intervention, *Srebp* sterol regulatory element-binding protein, *Ldl-R* LDL-Receptor, *Hmgcr* 3-Hydroxy-3-Methylglutaryl-CoA Reductase, *CYP7A1* cholesterol 7 alpha-hydroxylase, *GAPDH* Glycerinaldehyd-3-phosphate-dehydrogenase.

As CYP7A1, the key regulator of hepatic cholesterol and bile acid metabolism, was the most regulated protein among the proteomic screen and is also regulated by PGC1^28^ (Fig. 7A), we further addressed hepatic cholesterol metabolism at P21 and examined the transcription factors Srebp1 and 2 (Sterol regulatory element binding protein 1 and 2, Fig. 7B,E) and Ldl-R mRNA expression (LDL-Receptor, Fig. 7C). SREBP1 protein levels, as well as FAS-levels were significantly decreased in INT- offspring (Fig. 7E, *p* < 0.05 and Fig. 4E, *p* < 0.05). In contrast, *Srebp2* mRNA-expression levels revealed a significant increase in INT-offspring as well as a strong increase in *Hmgcr* (3-hydroxy-3-methyl-glutaryl-coenzyme A reductase) mRNA-levels (Fig. 7B,D).

## Discussion

In the present study, we determined the effects of ME on hepatic key mechanisms of lipid and glucose homeostasis in offspring early life at programming peak (P21): on the molecular level we found indications for (I) decreased intrahepatic “de novo*”* lipogenesis, (II) increased β-oxidation and mitochondrial biogenesis, (III) improved intrahepatic bile homeostasis and (IV) improved hepatic insulin signaling, while offspring of exercised dams were protected against HFD-induced weight gain and NAFLD in later life (P112).

The present data provide the first proteomic approach comparing the effects of ME in lean dams on liver function in offspring and demonstrate that ME may protect offspring from HFD-induced NAFLD by shaping the hepatic metabolism in early life.

Based on our preliminary data we identified the end of the lactation period (P21) as a critical window for developmental programming in which effects of maternal exercise are already detectable on a molecular level^11,29^. This is in line with previous studies which show that different conditions (maternal obesity, malnutrition or exercise) during pregnancy result in an altered offspring phenotype in later life, while molecular analysis reveal significant differences already during earlier stages of life^3,4,6,30,31^.

Therefore, proteomic analysis of liver tissue was initially performed at P21. They revealed as novel finding that CYP7A1, the rate-limiting enzyme transforming cholesterol to bile acid, as the main regulated protein between CO- and INT-offspring^32^. The significantly affected KEGG pathways in the proteomics screen were “metabolic pathways”, “steroid biosynthesis”, “PPAR signaling” and “oxidative phosphorylation”. Thus, important pathways involved in liver metabolism were induced only by ME. Complementary molecular analysis of liver tissue confirmed these strong effects on offspring’s hepatic lipid, glucose, cholesterol, and mitochondrial metabolism.

In detail, in early life at P21 hepatic AMPK signaling and ACC phosphorylation were significantly increased in INT-offspring, even though CO- and INT-offspring showed no obvious phenotypical differences at this time. AMPK acts as a major regulator of cellular and whole-body energy homeostasis coordinating multiple metabolic pathways^20^. One crucial AMPK function is the regulation of lipid metabolism through phosphorylation of ACC. This phosphorylation leads to an inactivation of ACC. ACC inactivation results in a decrease of malonyl-CoA activity and an increase of mitochondrial import of fatty acids and activation of fatty acid oxidation via upregulation of CPT1a^33^. In line with that, Cpt1a mRNA expression in INT offspring was increased at P21. Only recently, it has been shown that short-term strength training in obese mice increases CPT1a mRNA-levels^34^. However, the effect that ME alone can increase hepatic CPT1a-expression in exercised offspring has not been reported to date.

Phosphorylation of AMPK and ACC in offspring hepatic tissue indicates decreased lipogenesis, and CPT1a increase indicates augmented hepatic β-oxidation—a constellation that has been observed in INT-offspring in our study and has been shown to provide protection against the development of hepatic steatosis^18,35^.

**Figure 8:**
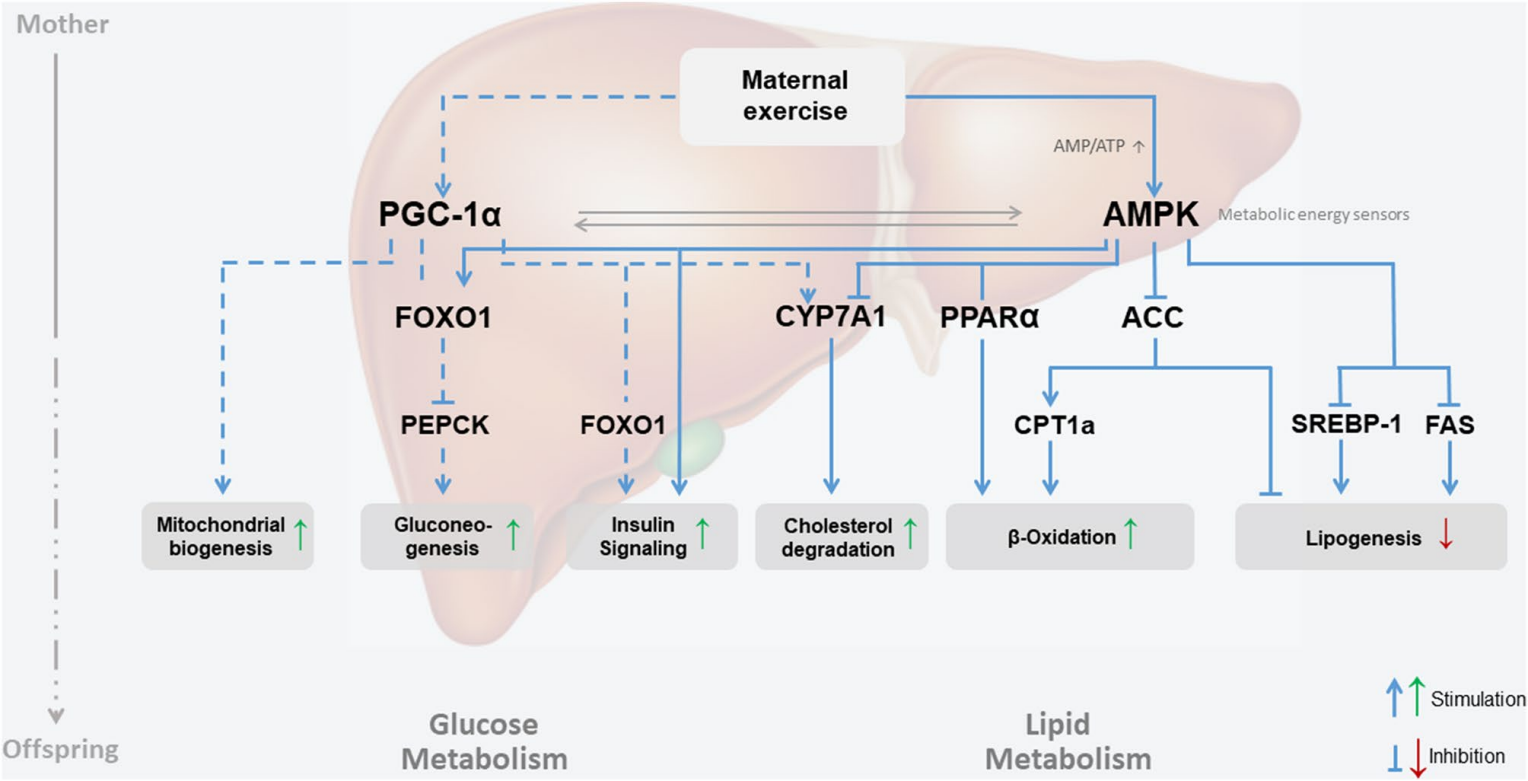
Hepatic metabolic programming—a working model depicting how maternal exercise during pregnancy conceivably affects hepatic metabolism of the offspring. Solid blue line: represent effects mediated by AMPK; dash blue line: represent effects mediated by PGC1α.

Another marker for hepatic β-oxidation which is regulated by AMPK is PPARα, which is the most abundant PPAR isotype in hepatocytes, was also increased in our INT-offspring at P21^23,36^. PPARα activation is known to improve steatosis and inflammation in pre-clinical models of NAFLD, whereas mice lacking hepatocytic PPARα develop hepatic steatosis and patients with NAFLD display reduced expression of PPARα^23,36^. Recently, PPARα/FoxO1 signaling pathway has been shown to play an important role in hepatic triglyceride metabolism by inhibiting triglyceride synthesis in “in vitro”- and “in vivo”-models^37^. Taken together, the effects of ME on AMPK-mediated hepatic PPARα action and ACC-Cpt1a axis in offspring might represent a potential key candidate metabolic pathway (Fig. 8).

Apart from lipid metabolism, insulin resistance is strongly implicated in the pathogenesis of NAFLD and also in disease progression from steatosis to NASH^38^. In line with the literature our results indicate for the first time an ameliorated hepatic insulin signaling via increased InsR, pAKT, Foxo1, PPARy, pAMPK and reduced SOCS3 level in younger offspring of exercised dams. Enhancement of AMPK-activity has been shown to improve insulin sensitivity^20^. Furthermore, AMPK is known for its effect to control gluconeogenesis via FoxO1 and therefore an increase of AMPK and FoxO1 in the liver is associated with reduced gluconeogenesis^18,20^. While our data indicate a clear activation of AMPK and FoxO1 in the liver of INT-offspring at P21, the expression of the rate-limiting gluconeogenic gene *Pepck* was, however, increased. These results are in accordance with recent findings of a study investigating exercise effects to obese mice resulting in increased glycogen levels^39^. Thus, the question arises whether elevated gluconeogenic pathways in INT-offspring may be part of a compensatory mechanism to avoid hypoglycemia during a phase of increased insulin sensitivity at P21 and further research is needed to clarify the effects of ME on offspring hepatic gluconeogenesis.

As a third metabolic aspect in this study, cholesterol and bile metabolism in the offspring turned out to be affected by ME during pregnancy, with CYP7A1 being the most prominently affected protein in the proteomics screen*.* Increased CYP7A1-expression, which can be induced by PGC1α, which was also upregulated in our study, is associated with significantly improved lipid homeostasis and protection against hepatic steatosis^16,28,32^. In line with our results, PPARy overexpression has also been shown to regulate transformation of cholesterol to bile acid via CYP7A1 in human hepatocytes^40^. As a novel finding, our results indicate hepatic CYP7A1 as an additional key metabolic factor in preventing NAFLD in offspring of exercised mothers. However, complementary analysis of bile acid metabolism and signaling including investigations of the bile acid-farnesoid X receptor-fibroblast growth factor 15 (bile acid–FXR–FGF15) axis are required to improve our understanding of the underlying mechanisms.

Apart from the aforementioned mechanisms enhanced activation of mitochondrial biogenesis has been also discussed to be associated with prevention of NAFLD^41^. Recent studies demonstrated that hepatic PGC1α-overexpression, which was also found in our INT-offspring at P21, results in increased hepatic fatty acid oxidation and reduced triacylglycerol accumulation and presumably protects against the development of hepatic steatosis^12,42–44^. Even though Tfam mRNA-expression did not differ between CO- and INT-offspring at P21, the observed Pgc1α upregulation, the activation of KEGG—pathway “oxidative phosphorylation” and the fact that Pgc1α methylation due to exercise were also observed in other organs prompted us to perform methylation analysis of the Pgc1α promoter region at P21. But in line with previous analysis from Laker et al., who only analyzed a single cytosine (corresponding to cytosine -235 in Supplementary Fig. 4A) we did not find any significant changes even though we examined more CpG-positions with a different method^45^. Thus, it is likely that other epigenetic modifications like histone modifications are responsible for the Pgc1α mRNA-expression alterations. For future experiments it would be interesting to perform methylation sequencing and chromatin immunoprecipitations for histone modifications to get a genome wide view on epigenetic alterations due to ME.

Taken together, we observed that ME creates a specific hepatic protein expression pattern during developmental programming involving numerous NAFLD-related enzymes at P21—already before any exposure to HFD-feeding happened—leading to the assumption that ME can indeed program offspring hepatic metabolism regarding protection against NAFLD while setting offspring hepatocytes into a “protective” metabolic state against hepatic fat accumulation. This hypothesis is further substantiated by the robust activation of hepatic AMPK-signaling in INT-HFD-offspring at P112 with concomitant improvement in histopathology. Thus, future interventions and pharmacological treatments could aim at preventing NAFLD by targeting mechanisms of perinatal hepatic programming.

Although there is currently no approved pharmacotherapy for NAFLD, pharmacological activation of AMPK and PPARα or pharmacological inhibition of its downstream target ACC represent attractive approaches to improve NAFLD^17,33,46^*.* A number of pharmacological studies focusing on direct or indirect activators of AMPK demonstrated that activation of AMPK can inhibit the intrahepatocellular de novo lipogenesis and increase fatty acid oxidation with concomitant lowering of the hepatic triglyceride content^17,35,47^. Moreover, first studies with oral ACC inhibitors showed a reduction of markers of liver injury and intrahepatic de novo lipogenesis, which resulted in reduced hepatic fat content^48^.

However, based on the complex pathophysiology and presumed multiple pathways affected in NAFLD, it is likely that combining therapies that engage different targets may provide a synergistic histopathologic benefit and represent the most efficient treatment regimes.

With our findings we want to underline the value of pharmacological targeting AMPK-ACC-CPT1a axis and spotlight CYP7A1 as an additional molecular target for pharmacological treatments^5,17,33,46^.

However, in contrast to these pharmacological approaches, our results also demonstrate that ME represents a promising non-pharmacological tool to prevent hepatic steatosis and NAFLD in offspring.

Even though translation from rodent studies to humans should be discussed critically, these results underline the importance of health programs for pregnant women, especially in those who suffers from metabolic disorders such as diabetes or obesity, and indicate the need for time- and cost-intensive long-term mother–child studies to examine the effects of ME to offspring hepatic metabolism in men.

Obesity rates in women of child-bearing age are rising worldwide and maternal obesity has been established as important risk factor for the development of NAFLD in offspring^49^. Since it is known that especially obese pregnant women don’t meet the current physical activity recommendations, a pharmacological treatment during the critical window of hepatic programming represents an alternative strategy to protect offspring metabolic health. As a first experimentally step, a treatment of obese pregnant mice with agonist of the AMPK-ACC-CPT1a axis or PPARα axis as well as their combination could be performed, to investigate whether this can combat the early origins of NAFLD in offspring and thereby contribute to prevent the global increase of NAFLD. A direct acting pharmacological CYP7A1 activator represents another promising approach and potential combination partner.

There are some limitations that should be considered when interpreting the results of this study: first, only male offspring was analyzed in our animal model. Some effects of developmental programming on long-term offspring health might be gender-specific^50^, but for cost and manpower reasons it was not possible to perform all phenotyping and molecular analysis for male and female offspring. However, since recently published studies examining the effects of ME on the offspring also used male offspring only, our data are well comparable^8,9,50^. The missing measurement of direct hepatocellular β-oxidation capacity remains a further limitation and should be conducted in future studies. Even though our data suggest that ME can prevent the detrimental effects on offspring metabolic health, the effects of paternal exercise on offspring hepatic metabolism cannot be completely excluded as male breeders also had short-term access to the running wheel during the mating process (48 h). While the offspring of exercised mothers in our study were mainly protected from simple steatosis, NASH as the more advanced proinflammatory phenotype with higher clinical and prognostic significance, could only be observed in a small proportion of mice. This might be due to the usage of HFD as dietary intervention in our study, which is often inadequate to induce NASH^51^. Since the dietary model we used is unable to reflect some of the most important histological features of NAFLD, a more suitable dietary model, e.g. a Western-Style-Diet, should be used in further studies. As Sheldon and colleagues, we used a voluntary wheel running program only during pregnancy in the present study^12^. For future studies it would be interesting to examine the effects of different exercise intensity and duration models to the offspring hepatic metabolism, because it seems likely that different levels of exercise intensity induce different effects in the offspring and that dose–response effects of different exposures to exercise should be considered exactly as particular stages of offspring development. Furthermore, non-fasted-serum levels should be critically discussed.

For the translation of our data we are in close cooperation with the German sports university. To examine long-term effects of ME on offspring hepatic health, a follow-up study of our known comparative cohort (see^14^) is planned.

### Conclusions

ME during pregnancy modulates offspring hepatic function via early hepatic programming while setting offspring hepatocytes into a “protective” metabolic state against the development of NAFLD by modulating five key mechanisms: I) decreased intrahepatic “de novo” lipogenesis via pAMPK and pACC, II) increased β-oxidation and mitochondrial biogenesis via PGC1α III) reduced intrahepatic triacylglycerol accumulation via pAMPK and PPARα IV) improved intrahepatic bile homeostasis via CYP7A1 and V) improved insulin resistance via SOCS3 (Fig. 8).

ME should therefore be considered for early prevention of obesity-related liver disease such as NAFLD.

## Methods

### Animal model

Our study was carried out by the Department of Pediatrics of the University Hospital of Cologne. The study was approved by the appropriate governmental authority (Institutional protocol number of the animal welfare application: AZ 8.87–50.10.37.09.292, Landesamt für Natur, Umwelt und Verbraucherschutz Nordrhein-Westfalen, Germany). All animal procedures were performed in accordance with the German Animal Welfare Law. Animal care and use was performed by qualified individuals, supervised by a veterinarian. The manuscript complies with the Animals in Research: Reporting In Vivo Experiments (ARRIVE) guidelines^52^.

Mice (C57BL/6N) were bred and held at the animal facility of the Department of Pharmacology of the University Hospital of Cologne (Cologne, Germany). Mice were housed in a room maintained at 22 ± 2 °C, exposed to humidity of 50–60% and a 12/12-h light/dark cycle. All breeding colonies were kept in individually ventilated cages (IVCs, Blue line cages type II long, Tecniplast, Italy). Three-week-old female mice were randomly assigned to a voluntary wheel running group (Maternal-INT, n = 9) or a control sedentary group (Maternal- CO_,_ n = 35) and were fed a regular standard laboratory chow diet (#R/M-H Ssniff, Germany; containing 412 g/kg carbohydrates, 190 g/kg protein, and 33 g/kg fat; total metabolizable energy 3220 kcal/kg, 9% of total metabolizable energy from fat) for 9–10 weeks during preconception, gestation and lactation period. While both groups (Maternal-CO and Maternal-INT) received the same standard diet, the running intervention group (Maternal-INT) had continuous access to a running wheel in their home cage during gestation (Fig. 1A). The running wheel was available from gestational day (G) 0 until G18, starting with mating. Running wheels were equipped with tachometers measuring distance (km), average speed (km/h) and time (h:m) as described before^11^. Male breeders were chow fed and had limited access to the running wheel during the mating process for 48 h. Body weight of the dams was monitored daily from G0 to G18. Blood samples for serum analysis were collected via submandibular puncture at gestational day G16. In order to minimize stress-induced side effects, dams were not fasted. Postpartum the body weight of both offspring groups (CO vs. INT, named after maternal conditions) was monitored daily starting immediately after birth at postnatal day (P) 1 (P1) up to P21. On P3, litter size was randomly adjusted to six for each litter. A subset of offspring was sacrificed at P21 and non-fasted blood samples were collected via intracardial puncture for further analyses. The remaining animals of CO and INT offspring continued on a standard diet until P70 (Fig. 1A). From P71 to P112, one-half of the CO- and INT-offspring was subjected to an obesogenic high-fat diet (#C1057 modified Altromin, Germany; containing 269 g/kg carbohydrates, 208 g/kg protein, and 351 g/kg fat; total metabolizable energy 5,237, 60% of metabolizable energy from fat), while the other half continued on standard chow. Consequently, four groups with distinct condition resulted from P70 to P112, termed as follows: CO, CO-HFD, INT and INT-HFD (Fig. 1A). After six week of HFD feeding, at P112, all animals were sacrificed and processed as described before^11,14^. All offspring were sacrificed via CO2-inhalation. Food intake measurement was performed in offspring at P70 when offspring were still on a standard diet and at P112 in offspring on a high fat diet (CO-HFD and INT-HFD, Supplementary Fig. 2F,G). From P21 to P112, animals were weighed weekly. For the duration of the whole study, all mice had free access to the experimental diets and water. To exclude gender influences, all studies were performed using male offspring only^6,8,50^. 1–2 pups per litter were studied at each time point.

### Analytical procedures

Blood samples for serum analyses were collected via submandibular puncture at G16 in dams. In offspring, blood samples were collected when animals were sacrificed via intracardial puncture. Blood samples were centrifuged for 10 min at 3.000 g and 4 °C and stored at -20 °C until analyzed.

### Intraperitoneal glucose tolerance test (ipGTT)

Animals were fasted for 16 h (18.00 h–10.00 h). After the determination of fasted blood glucose levels, each animal received an intraperitoneal injection of 20% glucose (10 mL/kg body weight = 2 g glucose/kg body weight). Blood glucose levels were measured after 15, 30, 60, and 120 min^53^.

### Biomarker analyses

Serum insulin and leptin levels were measured in a multiplex analyzer (Bio-Plex 200, Bio-Rad Laboratories, USA) according to the manufacturer’s instructions (Milliplex MAP, MA, USA). By using the median fluorescence intensity and the standard curve, the absolute concentration of each cytokine (pg/ml) was calculated (Bio-Plex Manager 6.1, Bio-Rad Laboratories).

### Quantitative real-time polymerase chain reaction (qRT-PCR)

Total ribonucleic acid (RNA) was isolated from liver using TRI-Reagent (Sigma-Aldrich) according to the manufacturer’s guidelines. RNA quantity and purity were determined by measuring UV absorption with a NanoDrop spectrophotometer (Nano Quant infinite M200 Pro). Quantitative changes in mRNA expression were determined by qRT-PCR as previously described^53^, using the 7,500 real-time PCR system (Applied Biosystems, Foster City, CA, USA). Primer pairs and Taqman probes are listed in Supplementary Table 1.

### Protein isolation

For protein isolation, samples were mixed with protein extraction buffer (6.65 mol/L Urea, 10% glycerol, 1% sodium dodecyl sulfate [SDS], 10 mmol/L Tris–HCl pH 6.8, 5 mmol/L Dithiothreitol, and 0.5 mmol/L phenylmethanesulfonylfluoride) and homogenized as previously described^53^. Concentration was measured using Bicichonin acid (BCA, Thermo Scientific).

### Immunoblotting

Immunoblotting of liver samples was performed as previously described^53^. Original blots are provided in Supplementary Fig. 6. Primary antibodies are listed in Supplementary Table 2. For detailed information see Supplementary Methods.

### Proteomics analysis

Protein isolation was performed as described above for western blot analysis. Following acetone precipitation, proteins were digested into peptides with trypsin, as previously described^54^. Peptides were purified using *SDB-RP* stage tips and stored at 4 °C prior analysis^55^. All samples were analyzed on a *Q-*Exactive Plus *(*Thermo Scientific*)* mass spectrometer that was coupled to an EASY nLC 1,200 UPLC *(*Thermo Scientific*)*, as previously described^54^. For MS library generation the raw files from data-dependent acquisition were processed with Maxquant (version 1.5.3.8) using default parameters. Briefly, MS2 spectra were searched against the Uniprot mouse.fasta (downloaded at: 16.6.2017) database, including a list of common contaminants. False discovery rates on protein and PSM level were estimated by the target-decoy approach to 1% (Protein FDR) and 1% (PSM FDR) respectively. Protein quantification was conducted using data-independent acquisition. Raw data were processed with Spectronaut Pulsar X (version 11) using default parameters, and data filtering was performed using the q-value option.

### Histological analysis of the liver tissue at P112

Upon sacrifice, liver tissue was excised and immediately fixed in 4% paraformaldehyde. Liver samples were embedded in paraffin and sectioned at 3 µm. Slices were stained with hematoxylin and eosin (H&E) for microscopic examination and the degree of steatosis, the type of steatosis, lobular inflammation and hepatocellular ballooning was evaluated. Additionally, sections were stained with Elastica-van-Gieson (EvG) for assessment of collagen deposition and fibrosis. To quantitatively assess the hepatic effect caused by exercise and dietary intervention, grade of steatosis, inflammation, ballooning and fibrosis were scored and NAFLD-activity score (NAS) was calculated as previously described^56–58^. The sections and the light microscopy were evaluated by one expert liver pathologist, who was blinded to the exercise and dietary conditions.

### Statistical analysis

The data are presented as mean ± standard error of the mean (SEM). The results of real-time RT-PCR were calculated based on the ΔΔ-Ct method and expressed as fold induction of mRNA expression compared with the corresponding control group (1.0-fold induction) as previously described^53^. For the phenotypical results at P112, we performed one-way ANOVA for non-parametric tests (Kruskal–Wallis-Test) followed by pair-wise Mann–Whitney t-tests if significance was stated (for nonparametric distribution). Body weight gain was calculated by a two-way ANOVA test followed by a Bonferroni post-test for repeated measures. For the comparison of only two groups (e.g. at P21), we performed pair-wise Mann–Whitney t-tests for nonparametric distribution. Statistical significance was defined as *p* < 0.05. The statistical analysis was performed with Graph Pad Prism 7.

See the Supplemental Material for further detail concerning methods.

## Supplementary information

Supplementary Information.

Supplementary Figure 1.

Supplementary Figure 2.

Supplementary Figure 3.

Supplementary Figure 4.

Supplementary Figure 5.

Supplementary Figure 6.

Supplementary Table 1.

Supplementary Table 2.

Supplementary Table 3.

Supplementary Table 4.

Supplementary Table 5.

## Data Availability

All data generated or analyzed during this study are included in this published article (and its Supplementary Information Files) and are available from the corresponding author on request.
