# Supplementary material for: Maternal exercise conveys protection against NAFLD in the offspring via hepatic metabolic programming
Source: Sci Rep. 2020 Sep 22;10:15424. doi: 10.1038/s41598-020-72022-6 (PMC7508970; doi:10.1038/s41598-020-72022-6)
Supplement: Supplementary file 1 — Supplementary Information. [file 41598_2020_72022_MOESM1_ESM.docx]

**Figure legends of Supplementary figures**

**Supplementary Figure 1:** Maternal phenotype. (A) Mean voluntary running distance (km/day) [INT (n=8)]. (B) Maternal body weight gain during pregnancy [CO (n=35), INT (n=7)]. (C/D) Maternal serum level of (C) insulin (ng/mL) and (D) leptin (ng/mL) at gestational day (G)16 [CO (n=7-9), INT (n=6)]. Mean ± SEM; *p < 0.05. Abbreviations: CO, control; INT, intervention; G, gestational day; ng, nanogram; mL, milliliter; P, postnatal day.

**Supplementary Figure 2:** Offspring phenotype. (A) Body weight at P21 [CO (n=52), INT (n=18)]. (B) Epigonadal fat pad weight at P21 [CO (n=31), INT (n=19)]. (C) Serum insulin level at P21 (ng/mL) [CO (n=7), INT (n=7)]. (D) Serum leptin level at P21 (ng/mL) [CO (n=7), INT (n=7)]. (E) Tfam mRNA expression at P21 [CO (n=4), INT (n=5)]. (F) Food intake 24h with standard diet at P70 [CO (n=9), INT (n=7)]. (G) Food intake 24h with HFD at P112 [CO (n=6), INT (n=5)]. (H) Serum insulin level at P112 (ng/mL) [CO (n=8), INT (n=7), CO-HFD (n=9), INT-HFD (n=5)]. (I) Serum leptin level at P112 (ng/mL) [CO (n=8), INT (n=7), CO-HFD (n=9), INT-HFD (n=5)]. Mean ± SEM; *p < 0.05, **p < 0.01, ***p < 0.001. Abbreviations: CO, control; INT, intervention; P, postnatal day; HFD, high fat diet; ng, nanogram; mL, milliliter; h, hours; Tfam, mitochondrial transcription factor A.

**Supplementary Figure 3:** Effects of maternal exercise during pregnancy on offspring AMPK-ACC signaling in later life (112). Protein expression of (A) pAMPK/AMPK, (B) pACC/ACC, (C) PPARα at P112. CO (n=5), INT (n=5), CO-HFD (n=5), INT-HFD (n=5). Mean ± SEM; Representative immunoblots are presented above the respective graph. *p < 0.05, **p < 0.01. Abbreviations: CO, control; INT, intervention; HFD, high fat diet; AMPK, adenosine monophosphate-activated protein kinase; PPARα, peroxisome proliferator-activated receptor alpha; ACC, acetyl-CoA carboxylase; GAPDH, Glycerinaldehyd-3-phosphate-dehydrogenase.

**Supplementary Figure 4:** Effects of maternal exercise on hepatic PGC1α promoter DNA methylation. (A) Schematic view of the *Pgc1α* promoter region and the CpGs present in a 1 kb region upstream of the transcriptional start site. Positions of the CpGs are given relative to the transcriptional start site of the transcript NM008904.2 (B) DNA methylation of the *Pgc1α* promoter. Shown is the percent methylation of the individual CpGs from 5 or 6 animals per group. The calculated bisulfite conversion rate was 98.1%. The given cytosine positions refer to the following positions in the mm10 genome build: -81, chr5:51,554,001; -118, chr5:51,554,038; -235, chr5:51,554,155; -424, chr5:51,554,344; -501, chr5:51,554,421; -670, chr5:51,554,590; -836, chr5:51,554,756. Mean ± SEM; *p < 0.05, Abbreviations: CO, control; INT, intervention; Pgc1α, peroxisome proliferator-activated receptor gamma coactivator 1-alpha; CpG, 5'-Cytosine-phosphate-Guanin-3' nucleotide sequence.

**Supplementary Figure 5:** Effects of ME on offspring hepatic metabolism at P112. Assessment of regulators of hepatic glucose and lipid metabolism by qPCR: (A) Pgc1α mRNA expression. (B) Tfam mRNA expression. (C) Srebp2 mRNA expression. (D) Hmgcr mRNA expression. (E) Cpt1a mRNA expression. (F) PPARγ mRNA expression. CO (n=7), INT (n=7), CO-HFD (n=6-9), INT-HFD (n=6-7). Mean ± SEM. *p < 0.05, **p < 0.01, ***p < 0.001. Abbreviations: CO, control; INT, intervention; HFD, high fat diet; Pgc1α, peroxisome proliferator-activated receptor gamma coactivator 1-alpha; Tfam, mitochondrial transcription factor A; Srebp, sterol regulatory element-binding protein; Hmgcr, 3-Hydroxy-3-Methylglutaryl-CoA Reductase; Cpt1a, carnitine palmitoyltransferase 1A; PPARγ, peroxisome proliferator-activated receptor gamma.

**Supplementary Figure 6:** Original uncropped images of immunoblots.

**Supplementary Material and Methods**

**Intraperitoneal glucose tolerance test**

Glucose tolerance test (GTT) was performed as previously described [1]. Animals were fasted for 16 h (1800 h–1000 h). After the determination of fasted blood glucose levels, each animal received an intraperitoneal injection of 20% glucose (10 mL/kg body weight = 2 g glucose/kg body weight). Blood glucose levels were measured after 15, 30, 60, and 120 min.

**Immunoblotting**

Immunblotting was performed as previously described [2]. Total protein of 30 µg was incubated with sample buffer (0.5 mol/L Tris, 20.6 % SDS, 25 % β-mercaptoethanol, 2.6 % bromphenolblue, and 10 % glycerin), incubated for 10 minutes at 70°C and then run on an SDS polyacrylamide gel electrophoresis. The gel was then transferred onto a nitrocellulose membrane (Whatman, Germany). After blocking the membrane with 5% milk and 2% bovine serum albumin in TBS-Tween (10 mmol/L Tris, 100 mmol/L NaCl, 0.1% Triton-X 100; pH 7.5), it was incubated with the first antibodies diluted in TBS-Tween at 4°C overnight. Antibodies used to detect specific targets are indicated in Supplemental Table 2. The horseradish peroxidase (HPO)-linked secondary antibodies goat anti-mouse HPO and goat anti-rabbit HPO (Cell Signaling, Germany) were used at 1:2000 dilutions at room temperature for 1 hour. After applying the ECL Prime Western Blotting Detection Reagent (GE Healthcare, United Kingdom) to the membrane, protein bands were acquired with the BIO-RAD Molecular Imager ChemiDOC XRS+ Imaging System (Bio-Rad Laboratories, Germany).

**Histological Analysis of the liver tissue at P112**

In detail, the amount of steatosis (percentage of hepatocytes containing fat droplets) was scored as 0 (<5 %); 1 (5-33%); 2 (≥33%-66%) and 3 (>66%). The type of steatosis was scored as 0 (none), 1 (microvesicular) or 2 (macrovesicular). Hepatocyte ballooning was classified as 0 (none), 1 (few ballooning, < twofold of the normal hepatocyte diameter) or 2 (prominent ballooning, > twofold of the normal hepatocyte diameter). Type of inflammation was scored as 1 (neutrophilic) and 2 (lymphocytic). Foci of lobular inflammation were scored as 0 (no inflammation foci), 1 (<2 inflammation foci per 200x field), 2 (>2 inflammation foci per 200x field). The amount of Mallory-Denk bodies was scored as 0 (none), 1 (rare) or 2 (many). In addition, NAFLD activity score (NAS) was calculated by using the sum of the of amount of steatosis, lobular inflammation and hepatocyte ballooning [3–5].

**Amplicon sequencing of bisulfite converted DNA**

500 ng of genomic DNA from liver were bisulfite converted using the EpiTect bisulfite kit (Qiagen, Hilden, Germany) according to the manufacturer´s recommendations. Subsequently, bisulfite converted DNA (BS-DNA) was subjected to PCR amplification for 35 PCR-cycles using the EpiTect methylation specific PCR (MSP) kit (Qiagen) following the manufacturer’s protocol. Oligonucleotides and annealing temperatures are given in Supplementary Table 5. PCR amplicons generated from one animal were pooled and purified using the Nucleospin Gel and PCR clean-up kit (Macherey-Nagel, Düren, Germany). Subsequently, amplicons were subjected to library preparation and sequencing on a NovaSeq6000 (Illumina, San Diego, CA, USA) at the Cologne Center for Genomics, University of Cologne. Amplicon-seq paired-end data were controlled for quality using FastQC v0.11.8 [6]. Overlapping mates within read pairs were merged into single sequences using PANDAseq v2.11 in pear mode and default parameters as all 7 generated amplicons were smaller in length than the sequencing length in total (2x 150 bp) [7]. Merged fastq files were used as input for bsmap v2.90 to map them against the region of interest from which the amplicons were amplified. Only those alignments were kept which had the correct start position according to their designed PCR product. Afterwards the methratios.py python script within the bsmap package was used to determine the methylation status of all Cs for all three methylation pattern types (CpG, CHG and CHH) [8]. The bisulfite conversion rate was calculated by the sum of C counts divided by the sum of CT counts over all CHG and CHH positions where H is any base but C. Graphical representation and statistical analyses were performed with GraphPad Prism 7 (GraphPad Software, La Jolla, CA, USA).

Sources:

[1] Bae-Gartz I, Janoschek R, Kloppe C, Vohlen C, Roels F, Oberthür A, et al. Running exercise in obese pregnancies prevents IL-6 trans-signaling in male offspring. Med Sci Sport Exerc 2016;48:829–38. doi:10.1249/MSS.0000000000000835.

[2] Rother E, Kuschewski R, Alcazar M, Oberthuer A, Bae-Gartz I, Vohlen C, et al. Hypothalamic JNK1 and IKKβ activation and impaired early postnatal glucose metabolism after maternal perinatal high-fat feeding. Endocrinology 2012;153:770–81. doi:10.1210/en.2011-1589.

[3] Brunt E, Janney C, Di Bisceglie A, Neuschwander-Tetri B, Bacon B. Nonalcoholic steatohepatitis: a proposal for grading and staging the histological lesions. Am J Gastroenterol 1999;94:2467–74. doi:10.1111/j.1572-0241.1999.01377.x.

[4] Kristiansen M, Veidal S, Rigbolt K, Tølbøl K, Roth J, Jelsing J, et al. Obese diet-induced mouse models of nonalcoholic steatohepatitis-tracking disease by liver biopsy. World J Hepatol 2016;8:673–84. doi:10.4254/wjh.v8.i16.673.

[5] Kleiner D, Brunt E, Van Natta A, Behling C, Contos M, Cummings O, et al. Design and validation of a histological scoring system for nonalcoholic fatty liver disease. Hepatology 2005;41:1313–21. doi:10.1002/hep.20701.

[6] Andrews S. A quality control tool for high throughput sequence data. n.d.

[7] Masella A, Bartram A, Truszkowski J, Brown D, Neufeld J. PANDAseq: paired-end assembler for illumina sequences. BMC Bioinformatics 2012;13:1–7. doi:10.1186/1471-2105-13-31.

[8] Xi Y, Li W. BSMAP: whole genome bisulfite sequence MAPping program. BMC Bioinformatics 2009;10:1–9. doi:10.1186/1471-2105-10-232.
