# Supplementary figures and images for: Maternal exercise conveys protection against NAFLD in the offspring via hepatic metabolic programming
Source: Sci Rep. 2020 Sep 22;10:15424. doi: 10.1038/s41598-020-72022-6 (PMC7508970; doi:10.1038/s41598-020-72022-6)

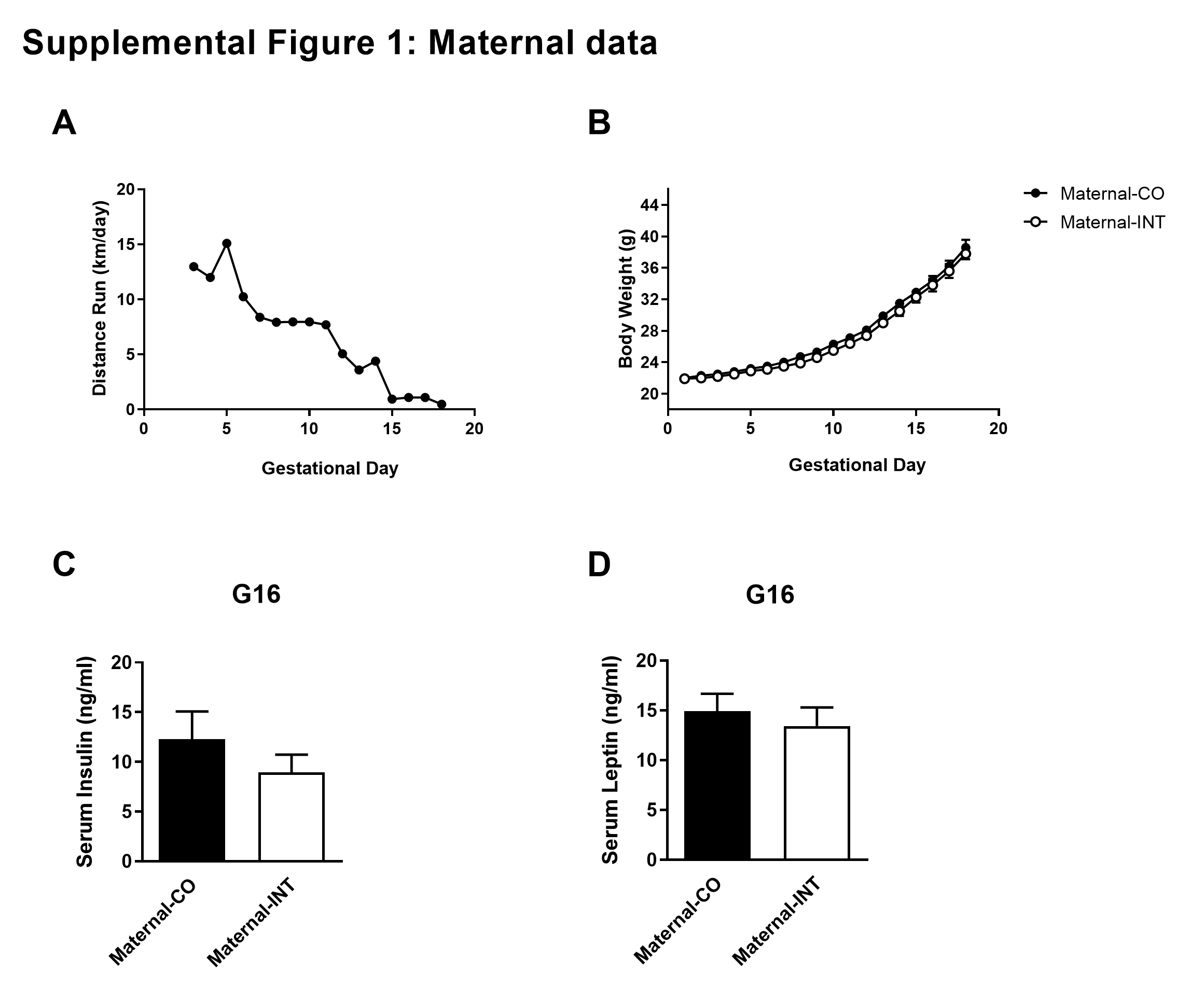

Supplement: Supplementary file 2 — Supplementary Figure 1. [file 41598_2020_72022_MOESM2_ESM.tif]

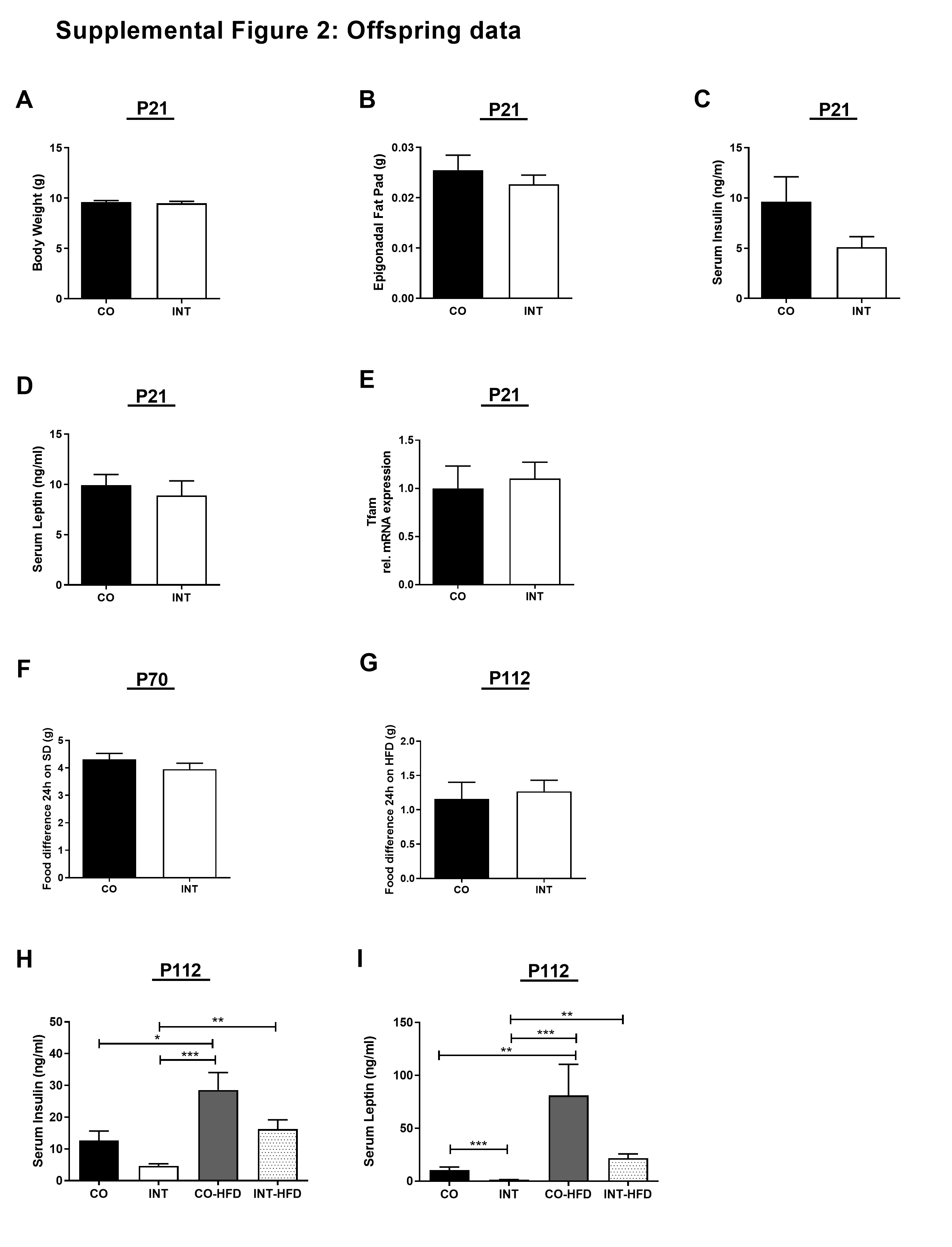

Supplement: Supplementary file 3 — Supplementary Figure 2. [file 41598_2020_72022_MOESM3_ESM.tif]

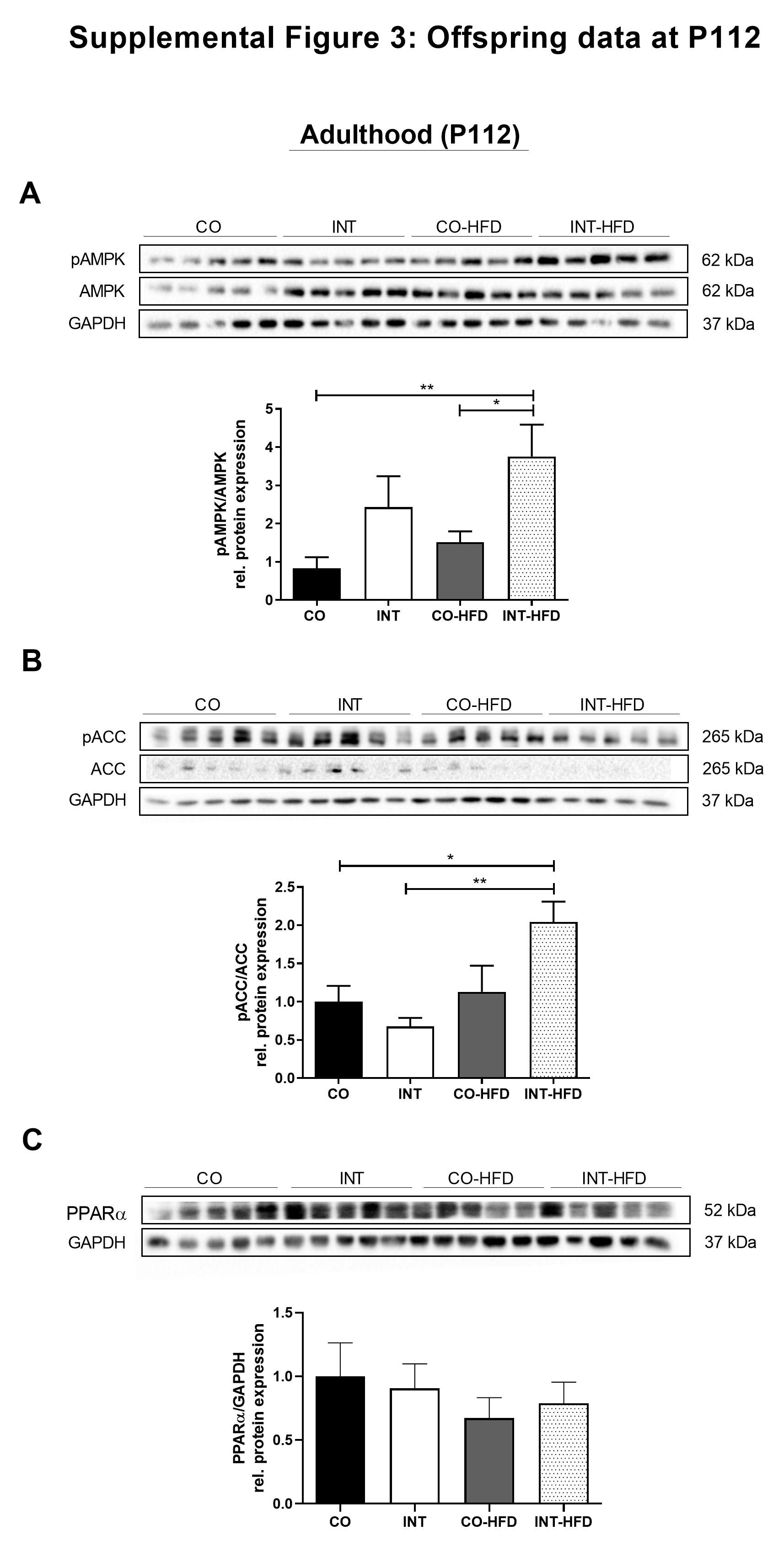

Supplement: Supplementary file 4 — Supplementary Figure 3. [file 41598_2020_72022_MOESM4_ESM.tif]

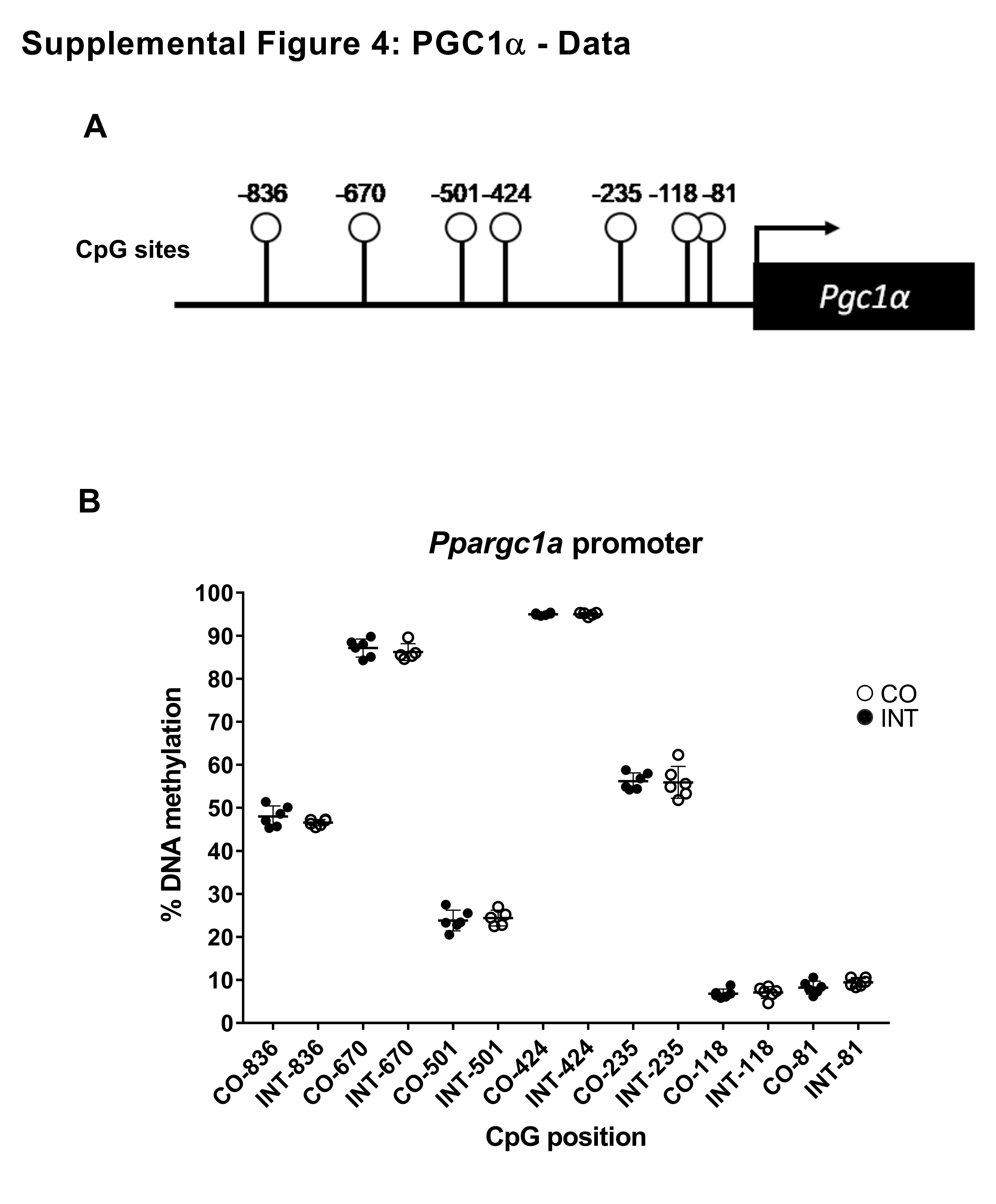

Supplement: Supplementary file 5 — Supplementary Figure 4. [file 41598_2020_72022_MOESM5_ESM.tif]

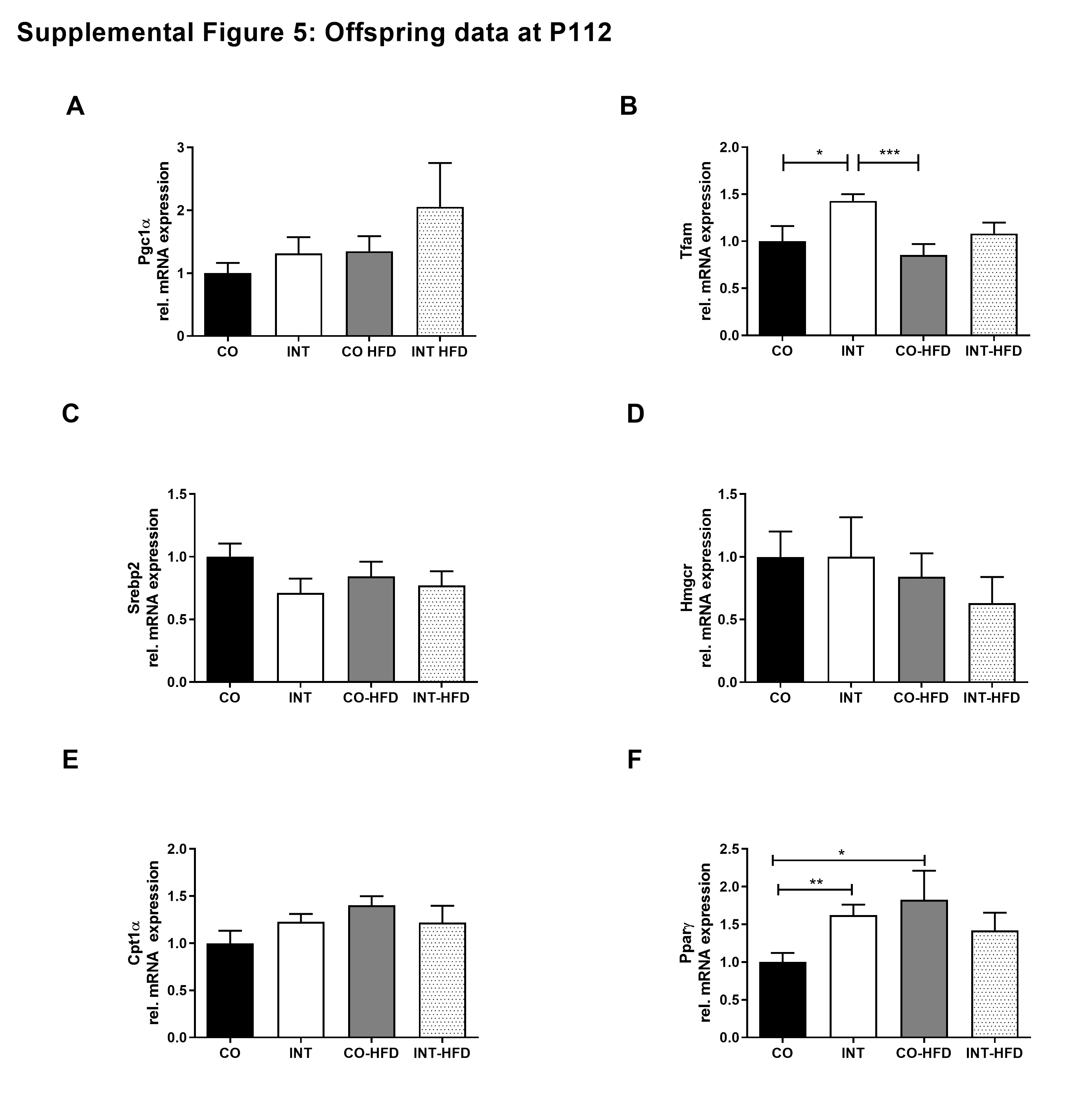

Supplement: Supplementary file 6 — Supplementary Figure 5. [file 41598_2020_72022_MOESM6_ESM.tif]
