# Supplementary material for: Maternal exercise conveys protection against NAFLD in the offspring via hepatic metabolic programming
Source: Sci Rep. 2020 Sep 22;10:15424. doi: 10.1038/s41598-020-72022-6 (PMC7508970; doi:10.1038/s41598-020-72022-6)
Supplement: Supplementary file 7 — Supplementary Figure 6. [file 41598_2020_72022_MOESM7_ESM.docx]

**Supplementary Figure 6**

**Title of manuscript:** Maternal exercise conveys protection against NAFLD in the offspring via hepatic metabolic programming

**Authors:** I Bae-Gartz, P Kasper, N Großmann, S Breuer, R Janoschek, T Kretschmer, S Appel, L Schmitz, C Vohlen, A Quaas, MR Schweiger, C Grimm, A Fischer, N Ferrari, C Graf, CK Frese, S Lang, M Demir, C Schramm, G Fink, T Goeser, J Dötsch, E Hucklenbruch-Rother

**Immunoblots**

Immunoblots blots represented in main Figure 5:

**A**

**B**

******

**C ****

Chemiluminescent detection of the immunoblots shown in the manuscript in (A) Figure 5A, (B) Figure 5B and (C) Figure 5C.

Immunoblots blots represented in main Figure 6:

**A**

**B**

**C**

***D***

Chemiluminescent detection of the immunoblots shown in the manuscript in (A) Figure 6C, (B) Figure 6D, (C) Figure 6E and (D) Figure 6F..

Immunoblots blots represented in main Figure 7:

**A**

Chemiluminescent detection of the immunoblots shown in the manuscript in (A) Figure 7E.
